# Signal Photon Extraction Method for ICESat-2 Data Using Slope and Elevation Information Provided by Stereo Images

**DOI:** 10.3390/s23218752

**Published:** 2023-10-27

**Authors:** Linyu Gu, Dazhao Fan, Song Ji, Zhihui Gong, Dongzi Li, Yang Dong

**Affiliations:** Institute of Geospatial Information, PLA Strategic Support Force Information Engineering University, Zhengzhou 450001, China

**Keywords:** ICESat-2, satellite stereo images, dense matching cloud, slope, DBSCAN, noise removal

## Abstract

Laser altimetry data from the Ice, Cloud, and land Elevation Satellite-2 (ICESat-2) contain a lot of noise, which necessitates the requirement for a signal photon extraction method. In this study, we propose a density clustering method, which combines slope and elevation information from optical stereo images and adaptively adjusts the neighborhood search direction in the along-track direction. The local classification density threshold was calculated adaptively according to the uneven spatial distribution of noise and signal density, and reliable surface signal points were extracted. The performance of the algorithm was validated for strong and weak beam laser altimetry data using optical stereo images with different resolutions and positioning accuracies. The results were compared qualitatively and quantitatively with those obtained using the ATL08 algorithm. The signal extraction quality was better than that of the ATL08 algorithm for steep slope and low signal-to-noise ratio (SNR) regions. The proposed method can better balance the relationship between recall and precision, and its F_1_-score was higher than that of the ATL08 algorithm. The method can accurately extract continuous and reliable surface signals for both strong and weak beams among different terrains and land cover types.

## 1. Introduction

Light detection and ranging (LiDAR), together with spectral imaging technology and synthetic aperture radar technology, is regarded as a core information acquisition technology for Earth observation [1]. The Ice, Cloud, and land Elevation Satellite-2 (ICESat-2) is the first Earth observation satellite equipped with a photon-counting laser altimeter system, the Advanced Topographic Laser Altimeter System (ATLAS), which adopts a sampling strategy with unprecedented spatial details [2]. It is highly sensitive, capable of micro-pulse and high-repetition-rate detection, and can continuously detect the surface profile in detail, rendering it highly applicable in polar ice sheet monitoring [3,4,5,6], forest biomass estimation [7,8,9,10], lake level monitoring [11,12,13,14], and ocean observation [15,16,17,18]. However, the low energy of the laser pulses emitted by the system renders it susceptible to factors such as surface reflectance, atmospheric scattering, solar radiation, and detector dark counts, resulting in noisy data [19], which, to a certain extent, limits the scientific application of ICESat-2.

Several methods, such as raster image processing [20,21], local statistical parameters [22,23], and spatial density clustering [24,25,26], have been proposed to effectively reduce the noise associated with photon-counting laser altimeter data and accurately extract surface signal photons. The laser altimeter system typically generates more signal photons from the ground surface, which are of higher density than noise-associated photons. The density-based method is therefore commonly used in ground signal photon detection. Zhang et al. [24] improved the density-based spatial clustering of applications with noise (DBSCAN) algorithm to detect signal photons from photon-counting LiDAR measurements; this method achieved good results for plains but was not effective in detecting highly fluctuating terrain. An ellipse with adaptive direction [26,27,28,29] was therefore adopted, and the size of the ellipse was adjusted according to the terrain slope [29,30] to obtain adjacent signal photons from different terrain conditions as accurately as possible, which led to good signal detection results. In addition, the spatial distribution differences associated with surface reflectance, terrain fluctuation, atmospheric conditions, and solar zenith angle mean that the photon density varies greatly along the orbital direction and the global density threshold has certain limitations. It is therefore necessary to dynamically adjust the density threshold according to the along-track slope or signal photon density differences [26,29].

In general, four key parameters—neighborhood search shape, size, search direction, and density threshold—need to be improved, regardless of the density-based method used, with the search direction being particularly difficult to determine accurately. The existing methods adaptively adjust the search direction by estimating the surface slope using the rough denoising results [30] or estimating the surface slope by traversing multiple directions to calculate the density extreme value [27,29]. However, these methods are not particularly effective in areas with a complex terrain, especially for weak beam data with a low signal-to-noise ratio (SNR). Zhang et al. [26] solved this problem by using strong beam-derived ground signal photons to fit the relationship between the slope and noise, allowing slope estimation from the weak beam data so that the neighborhood search direction could be adaptively adjusted, and achieved good detection results for mountainous areas. High-precision point cloud data and digital elevation models (DEMs) can be obtained by the dense matching of high-resolution optical satellite stereo images [31,32], from which reliable topographic slopes can be calculated to provide a reference for the neighborhood search direction of signal photons, improving the extraction accuracy of said signal photons.

In this study, we propose an adaptive signal photon extraction method that integrates slope information with the elevation information extracted from optical stereo images, named the Slope, Elevation, and Density Based Spatial Clustering of Applications with Noise (SE-DBSCAN). First, a mathematical model of the along-track terrain slope was established and extracted from the dense matching point cloud of the optical stereo images according to the track position information of the laser altimetry data. Then, according to the terrain similarity theory, a registration strategy based on elevation differences registered the dense matching point cloud and laser altimetry data to solve the problem of inconsistent positioning accuracy between the optical satellite images and the laser altimetry data. Finally, a slope optimization strategy adaptively adjusted the search direction of the clustering algorithm, with the local threshold used to reduce the influence of unevenly distributed photon data density in the along-track direction.

## 2. Materials and Methods

### 2.1. Research Area and Datasets

Zhengzhou in central China was selected as the study area (112.50–113.50° E, 34.15–34.60° N). The terrain in this area varies considerably, with mountains, hills, plains, and an appropriate amount of vegetation and buildings distributed around the study area. The surface slopes differ appreciably, meaning that the performance of the algorithm can be fully verified. The method proposed in this study was verified and analyzed using optical stereo images from Gaofen-7, Ziyuan-3, and Tianhui-1, as well as data from the ATL03 [33] and ATL08 [34] products in the ICESat-2/ATLAS laser altimeter system. Table 1 describes the main parameters of the images in the study area. 

**Figure 1:**
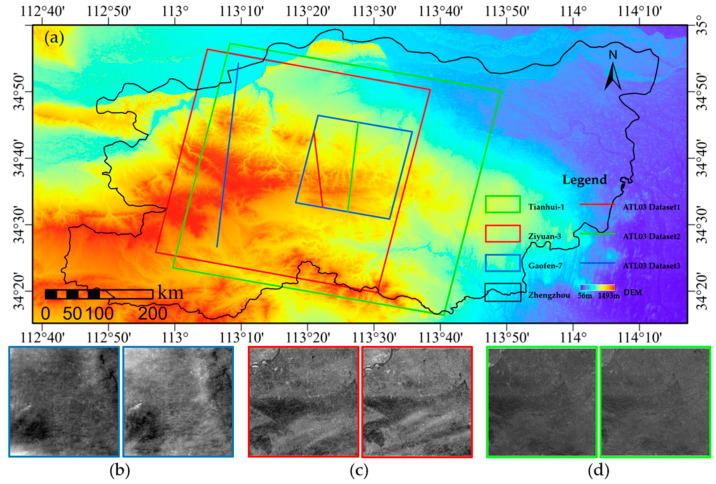
Study area and data. (**a**) Schematic diagram showing the laser measurement and optical satellite stereo images. Blue, red, and green rectangles denote the coverage of Gaofen-7, Ziyuan-3, and Tianhui-1, respectively. Blue, red, and green lines represent the spatial distribution positions of the laser altimetry data. Red and green lines represent strong beam laser altimetry data, while blue line represents weak beam data. (**b**–**d**) The optical stereo images of Gaofen-7, Ziyuan-3, and Tianhui-1, respectively.

To minimize the influence that changes in the terrain and land cover types had on the experiment, the laser altimetry data for the study area were screened with the condition of being as consistent as possible in terms of acquisition time or season. Laser altimetry data from different seasons were also selected to fully verify the accuracy of the slope extraction from different types of optical stereo images. The spatial distribution of the optical stereo images and laser altimetry data in the study area is shown in Figure 1 and the data are described in Table 2. The coverage area for this dataset is mainly mountainous with large terrain fluctuations and an appropriate amount of vegetation and buildings.

### 2.2. Methods

**Figure 2:**
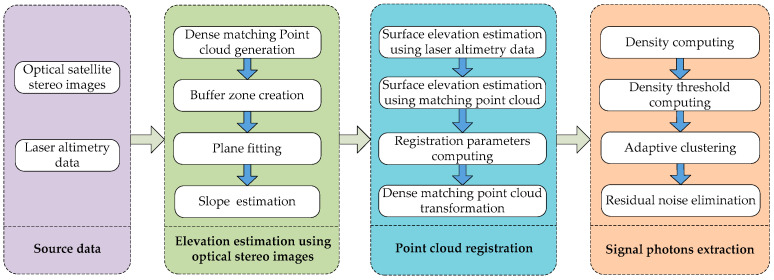
Flowchart of the proposed method.

First, dense matching of the optical stereo images was performed to obtain point clouds with which to invert the surface slope and elevation at the location of the laser altimetry data. The dense matching point cloud was then matched with the laser altimetry data. Finally, the optimal terrain matching slope was selected from the terrain slope estimated using the dense matching point cloud as reference data for adaptive signal and noise identification. A flowchart of the overall proposed method is shown in Figure 2.

#### 2.2.1. Slope Estimation Using Optical Stereo Images

**Figure 3:**
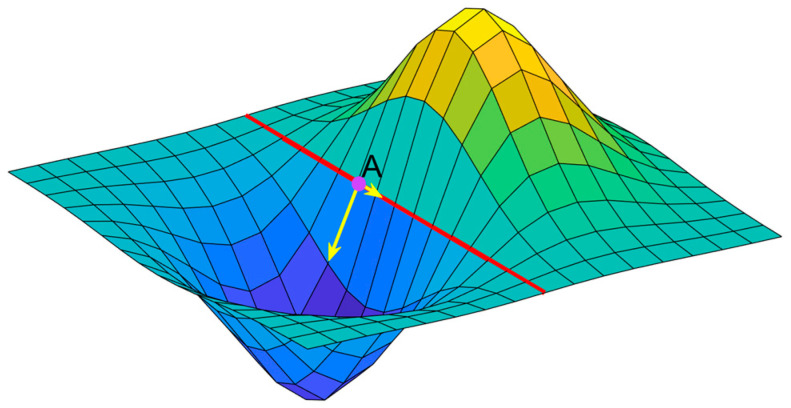
Schematic diagram of the difference between the terrain slope and the along-track terrain slope. The colored pixels of the terrain correspond to different elevations. The red line represents the ground track of the ATL03 data, and the size and direction of the two yellow arrows correspond to the slope gradient and direction of the terrain and the along-track terrain, respectively. Point A is an example of where the slope can be both steep and shallow depending on the track direction.

The ATL03 product generates terrain surface profile data with a large amount of noise; thus, the terrain slope in the along-track direction can be used as reference data to extract signal photons from the ATL03 data. However, it is necessary to first obtain information about the elevation along the track. The semi-global matching (SGM) [35] method is used to obtain a point cloud for a region of interest via the dense matching of optical stereo images. It should be noted that, when calculating the reference terrain slope in the along-track direction, the slope that is estimated from dense matching point cloud data should not be taken directly as the reference slope because large slope estimation errors will be introduced when the slope aspect differs from the track direction. In Figure 3, the terrain at point A is steep and the slope is large; however, the ground track direction of the ATL03 data is parallel to the slope at this position and close to 0°, which differs considerably from the actual terrain slope.

**Figure 4:**
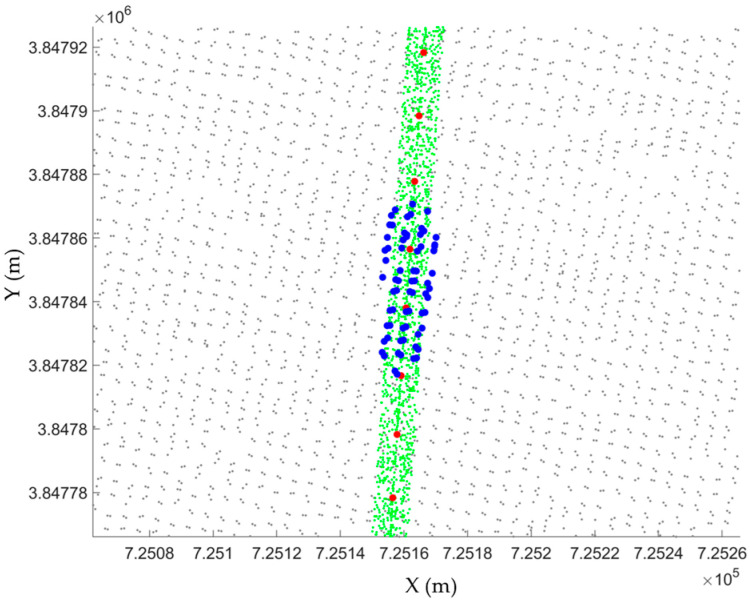
Dense matching point clouds within buffer zones. The green, gray, blue and red points denote the ATL03 data, the dense matching point clouds, the dense matching point clouds in a buffer zone, and the center points of the fitting centerlines of the ATL03 segments, respectively.

A fitting plane-based along-track terrain slope estimation method was therefore proposed to accurately estimate the along-track terrain slope in the ATL03 data. Three main steps were involved in developing the algorithm. First, the ATL03 data were divided into segments at intervals of a regular distance, with some overlap. The corresponding latitudes and longitudes and the centerline of a segment could then be obtained according to the photon data of the segment. Next, a buffer zone extending a certain length from the centerline was created for each segment. The three-dimensional coordinates of the dense matching point cloud inside the buffer zone were acquired, and the fitting plane was calculated. Finally, the intersection line between the vertical plane on which the fitting centerline of the segment was located and the fitting plane of the dense matching point cloud was calculated. The slope of the line was thus the along-track terrain slope in the ATL03 data within the segment. In Figure 4, the coordinates of the center points were calculated using Equation (1) for the centerline:

(1) y=kx+b

where k denotes the slope of the line and b is the intercept of the line on the y-axis. The equation by which the vertical plane upon which the centerline lies was calculated using Equation (2):

(2) kx−y+b=0

where k and b have the same meaning as in Equation (1). To estimate the along-track slope of a segment, the dense matching point cloud data (blue points in Figure 4) in the buffer zones were first obtained. Based on their three-dimensional coordinates, a fitting local terrain plane was calculated, as expressed by Equation (3):

(3) Ax+By+Cz+D=0

where (A, B, C) denotes the normal vector of the local terrain plane. Next, the equation of the vertical plane at which the centerline was located and that of the fitting local terrain plane was used to calculate the intersection line, as follows:

(4) $$\frac{x-x_{0}}{m}=\frac{y-y_{0}}{n}=\frac{z-z_{0}}{l}$$

where (m, n, l) is the directional vector and $\left(x_{0},\;{\;y}_{0},\;{\;z}_{0}\right)$ is a point on the line. The along-track slope (φ) of the segment was calculated using Equation (5):

(5) $$\varphi =arctan(\frac{l}{\sqrt{m^{2}+n^{2}}})$$

where the definitions of m, n, and l have the same meaning as in Equation (4). 

**Figure 5:**
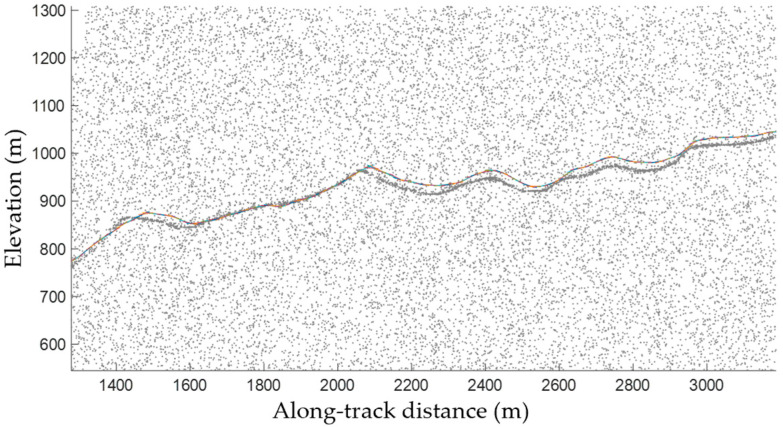
Overlay of the ATL03 data and slopes estimated using dense matching point clouds. The grey points and colored lines denote the ATL03 data, and the slopes estimated using dense matching point clouds of the ATL03 segments, respectively.

Figure 5 shows the fitting terrain slopes in each data segment, which were obtained by setting the segment length, step size, and buffer distance to 40, 10, and 17.5 m (equal to the laser spot diameter), respectively. The figure demonstrates significant consistency between the terrain slopes that were estimated based on dense matching point cloud data and the actual terrain slopes, indicating that the estimated results can accurately reflect the topography.

#### 2.2.2. Registration of Dense Matching Point Cloud Data to Laser Altimetry Data

The point cloud data generated by the dense matching of optical satellite stereo images using the initial orientation parameters may be less accurate in terms of horizontal and vertical positioning. Although laser altimetry data have relatively high elevation accuracy, the horizontal positioning accuracy is comparatively low [2,36]. The along-track terrain slope fitting method in Section 2.2.1 therefore cannot truly reflect the terrain slope. As revealed in Figure 3, the derived along-track terrain slopes and elevations differed from the laser altimetry data in terms of both horizontal position and elevation. Hence, if the fitting results are to be used for subsequent calculation, the dense matching point cloud data must first be correlated with the laser altimetry data. A strategy based on the elevation differences of the profiles was therefore designed in accordance with the terrain similarity theory to match the dense matching point clouds with the laser altimetry data. The steps-wise breakdown of the strategy is as follows:

Step 1: The elevation of the point at which the densest laser altimetry point cloud was observed was calculated for each segment.

**Figure 6:**
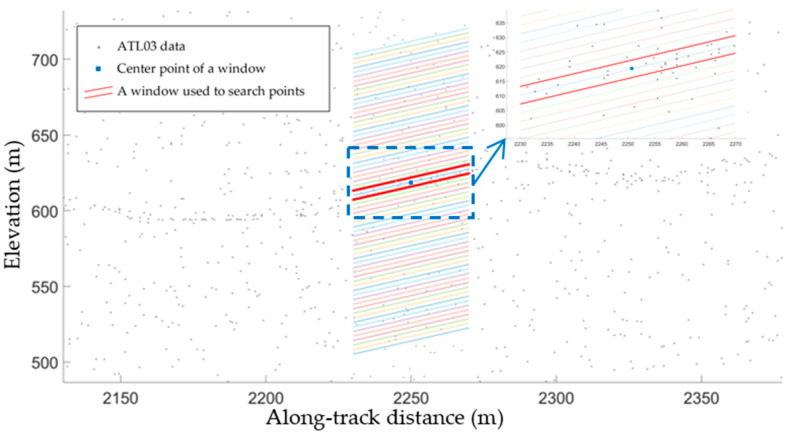
Laser altimetry point cloud density and terrain elevation estimation based on window division, showing a divided segment and its enlarged view of the dashed blue rectangle area. Each window bounded by two lines with the same color has a different color. Since there is some overlap between the two adjacent windows, and the windows are drawn from top to bottom, the lower border of a window is covered by another window with a different color.

Theoretically, when the density of the potential laser altimetry point cloud is estimated along the terrain slope, the highest density is obtained. Therefore, neighborhood search along the terrain slope is critical for accurate estimation of the highest point cloud density in each segment. Nevertheless, spatial distribution differences were observed between the segment-wise derived slopes and elevations in Section 2.2.1 and the actual terrain. Hence, the along-track slope values of the laser altimetry data had to be carefully selected in order to calculate the maximum point cloud density in each segment. First, a segment was divided into sub-segments. The window length was identical to that of the segment, while the window width had to be selected carefully. A small window can result in a large number of calculations, meaning that the relationships between the terrain features may be lost and the calculation results rendered inaccurate. On the contrary, significant noise can be included when a large window is used, meaning that the calculated densities will be lower. In short, an appropriate window width, usually around 2–3 m, needs to be chosen. Important factors to be considered are the elevation accuracy of the laser altimetry data, the land cover type, and the position and accuracy of the derived slopes based on the dense matching point clouds. In addition, to ensure the accuracy of the density estimation, some parts of the sub-segments should overlap. In other words, the step size should be smaller than the window width, and the angle of rotation of the window should be as consistent as possible with the actual slope. A segment was divided into sub-segments according to the dense matching point cloud-fitting slope and elevation. The angle of rotation for the window followed the calculated slope of the segment of interest and the estimated slopes of several segments adjacent to the segment of interest. The maximum densities of all the sub-segments were then calculated. The terrain slope corresponding to the highest point cloud density was likely closest to the actual terrain slope, and this elevation was thus taken as the terrain elevation for the segment. This process was repeated until the terrain elevations for all segments were calculated. Figure 6 shows that the angle of rotation followed the best-fitting slope based on the dense matching point cloud of the segment. The highest average density of the laser altimetry point cloud lies between the two red solid lines. The center point (blue point) in the window bounded by the two lines denotes the terrain elevation of the segment based on the ATL03 data.

Step 2: The elevation of the dense matching point cloud data at the center point of each segment was extracted using the segment length and step size selected in Step 1. The elevation at the center of a segment was interpolated using the fitting terrain line based on the dense matching point cloud data.

Step 3: Outliers from the terrain elevations derived from the laser altimetry data and dense matching point clouds were eliminated. Standard deviations for the elevation differences at the center of the segments were calculated. Data that lay outside the 3*σ* ranges were discarded.

Step 4: The registration parameters were obtained.

The elevation data obtained were utilized to generate fitting spline curves, which were then translated using the fixed along-track distance moving step size and vertical moving step size, with a new central elevation interpolated from the curve after each translation. The standard deviations for the differences in the center elevations obtained from the laser altimetry data and the dense matching point cloud process were then calculated for all segments. The along-track translation distance and vertical moving distance with the smallest standard deviation were selected as the registration parameters, meaning that the horizontal registration parameter could be considered the along-track horizontal translation parameter. Next, according to the orbit orientation, the horizontal translation parameter was decomposed into x- and y-axis translation parameters, from which the planar positions and elevations of the dense matching point clouds could be adjusted and the point clouds registered to the laser altimetry data.

**Figure 7:**
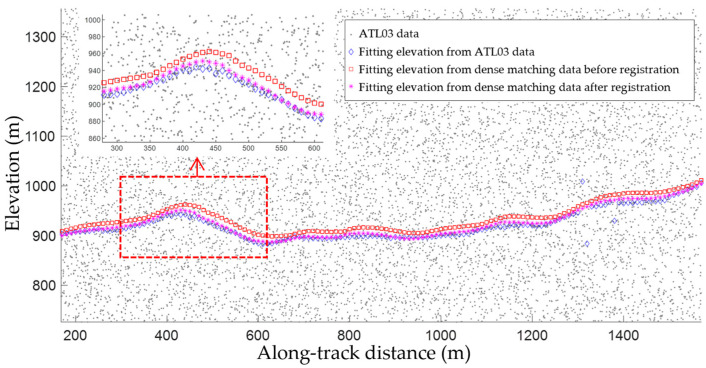
Point registration based on terrain similarity. Gray dots represent the ATL03 data, while blue diamonds denote the surface elevations obtained from the laser altimetry data. The orange squares are the along-track terrain elevations based on the dense matching point clouds and the purple stars represent their matched counterparts. The top left corner shows the enlarged registration result in the dashed red rectangle.

Figure 7 shows the results of registering the dense matching point clouds to the laser altimetry point clouds. It should be noted that most terrain elevations could be accurately estimated based on the ATL03 data using the maximum local density method. However, the random noise distributions meant that abnormally high values were observed, as shown in the right of the figure. Eliminating the outliners (Step 3) allowed better registration results to be obtained.

#### 2.2.3. Adaptive Density Clustering Algorithm Based on an Optimally Fitting Terrain Slope

The DBSCAN algorithm, which was first proposed by Easter [37], is a classical density-based clustering algorithm in which closely packed points are grouped into a cluster when the density reaches a desired value. The main parameters of the DBSCAN algorithm include the neighborhood shape and size, the search direction, and the density threshold. These parameters are critical to the algorithm’s performance. Elliptical neighborhoods can often better reflect the actual densities of potential signals [24,25,26,27,28,29,30]. Therefore, the SE-DBSCAN algorithm mainly focuses on the semi-major and semi-minor lengths of the elliptical neighborhood, the search direction (the inclination angle of the semi-major axis), and the density threshold.

The semi-major and semi-minor lengths of the elliptical neighborhood are related to factors such as the laser spot size, sampling frequency, laser energy, and the densities of the signal and noise points. Appropriate semi-major and semi-minor lengths should conform to the continuity of the surface data points. Detectors can sense reflected photons anywhere along the track. In addition, it has previously been pointed out that terrain elevations obtained via 50-beam pulse inversion can better reflect the detailed characteristics of the ground surface [38]. Because dense matching point clouds can provide relatively accurate along-track terrain slopes, a reasonably long semi-major length was selected to avoid noise interference in this study. If the semi-minor length is too long, more noise points will be included and the local densities will be underestimated. By contrast, if the semi-minor length is too short, the relationships between the ground surface points will be lost. Hence, the length was set to include all ground surface signal photons.

The search direction, which is the angle of rotation of the elliptical neighborhood, could be expressed by the along-track slope estimation based on the dense matching point cloud according to the method described in Section 2.2.2. After correlation, the terrain obtained from the dense matching point cloud data became more consistent with the potential signal points of the laser altimetry data. Yet, some differences remained. An optimal selection strategy had to be designed to select the best-fitting slope with respect to the ATL03 terrain from those obtained using data from the dense matching point cloud process. The slope derived for the segment corresponding to the signal point of interest and those of several adjacent segments were adopted as the search directions and a horizontal component was added (especially when extracting signals in urban areas). The highest density among these search directions was considered as the density at the point of interest, while the slope corresponding to the highest density was assumed to be the optimal search direction.

**Figure 8:**
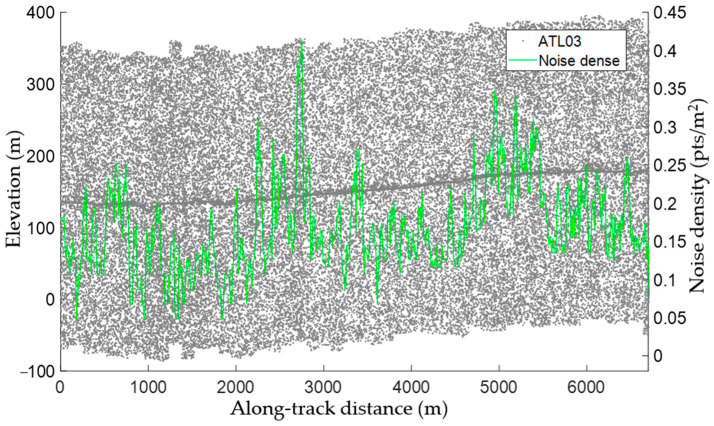
Along-track noise density distribution (green line) and the ATL03 data (gray dots).

The density threshold is that at which the noise and signal points are classified. The value of this threshold is related to the densities of the noise and signal points and the range of the search neighborhood and can be set according to the estimated noise and signal point densities and the size of the previously determined neighborhood. The density threshold should lie between the noise and signal density for a potential signal region, and to better preserve the ground surface signal points, the density threshold should be closer to the maximum noise density. Furthermore, due to differences in factors such as surface reflectance, atmospheric environment, altitude, and sun incidence angle, the noise density varies significantly along the track direction [39] and the signal point density is uneven. The fluctuations observed in the noise density distribution (Figure 8) suggest that the along-track distribution of the noise was uneven, with considerable spatial differences. The noise density corresponding to the curve was calculated using noise photons; the surface elevations obtained from the matched dense matching point clouds were taken as references and points with higher or lower values were considered noise. The noise density was then calculated segment-wise. It was apparent that the density threshold associated with along-track signal photon extraction was not global and that a local density threshold was required for each segment.

The Otsu method was employed to calculate the local density threshold of each segment. Based on the point cloud density for a segment, the optimal division threshold was calculated, allowing the variance between signal and noise photons to be maximized. The local density threshold of each segment was then calculated using Equations (6)–(8):

(6) $$w_{0}\left(t\right)=\frac{t}{n}$$

(7) $$w_{1}\left(t\right)=1-\frac{t}{n}$$

(8) $$\sigma^{2}=w_{0}{\left(t\right)w}_{1}{\left(t\right)\left(u_{1}\left(t\right)-u_{0}\left(t\right)\right)}^{2}$$

where *n* denotes the total number of photons in each segment; t is the number of signal photons; $w_{0}\left(t\right)$ represents the proportion of signal photons to the total number of photons in each segment; $u_{0}\left(t\right)$ is the average signal photon density; $w_{1}\left(t\right)$ denotes the proportion of noise photons to the total number of photons in each segment; $u_{1}\left(t\right)$ is the average noise photon density; $\sigma^{2}$ is the inter-class variance, which is maximized when *t* reaches the optimal threshold.

It should be noted that, in the case of complex terrains or a low SNR, the signal point densities of some segments were low, and the noise densities were high. The local threshold calculated using the Otsu method may be higher than that obtained for the point densities in the signal regions. It is therefore necessary to define upper and lower limits for the density threshold. The maximum density of each segment was taken as the upper limit, and the lower limit was the maximum noise density for each segment. When the density threshold calculated using the Otsu method was higher than the maximum density within a segment, the latter was chosen as the density threshold for the segment. Meanwhile, if the calculated threshold was smaller than the maximum noise density, the average was adopted as the density threshold so as to include as many signal points as possible.

In this situation, some high-density noise may be retained as signal points. Hence, more noise must be removed. In this study, the residual noise was eliminated based on the absolute elevation difference and the 3σ confidence level. First, the extracted signal photons were separated into segments of a fixed length, and points with elevations higher or lower than the values obtained from dense matching point clouds were discarded. The average values and standard deviations of the elevations associated with the remaining photons in each segment were then calculated. If the elevation of a point differed from the average elevation by more than 3σ, the point was considered noise and was removed from the results.

#### 2.2.4. Evaluation Metrics

To examine the denoising accuracy of the proposed algorithm and compare the resulting data with the ATL08 data, optical images were combined with manual inspection and the ATL08 classification results to locate the actual ground surface points associated with the data. These points were then used as reference data for algorithm evaluation. Laser altimetry data are discrete point cloud data that describe surface profiles. Hence, in evaluating the target points, those in the neighborhood of the target point were extracted from the reference data and the point closest to the target identified. Its class (noise or signal) was then compared with that of the target and the recall, precision, and F_1_-score calculated to evaluate the performance of the algorithm [26] using Equations (9)–(11):

(9) $$R=\frac{TP}{TP+FN}$$

(10) $$P=\frac{TP}{TP+FP}$$

(11) $$F_{1}=\frac{2P\times R}{P+R}$$

where *R*, *P*, and *F*_1_ denote the recall, precision, and *F*_1_-score, respectively; true positive (*TP*) is the number of correctly identified signal photons; false negative (*FN*) represents the number of photons identified as noise photons that are, in fact, signal photons; false positive (*FP*) is the number of photons identified as signal photons that are, in fact, noise photons. *TP* means the signal photons belong to the terrain surface in the real world and the extraction result is correct, *FN* means that the signal photons belong to the terrain surface but the extraction result is wrong, and FP means that the noise photons not in the terrain surface area are extracted as a signal.

## 3. Results and Discussion

### 3.1. Accuracy of the Terrain Slopes Obtained from Inversion Using Stereo Optical Images

**Figure 9:**
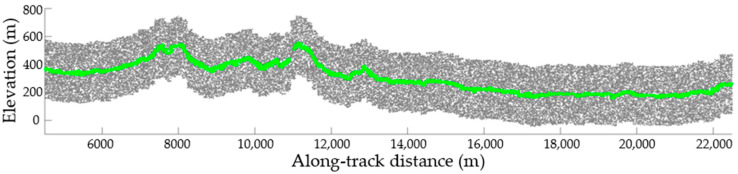
Laser altimetry data describing the 4.5–22.5 km segment in dataset 1 for ATL03 (gray dots) and ALT08 (green dots).

**Figure 10:**
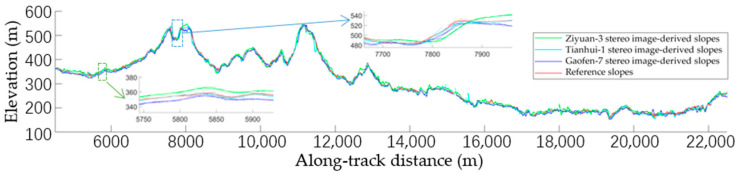
Terrain elevations and slopes obtained from different optical stereo images and the reference laser altimetry data. Terrain elevations and slopes in the dashed green and blue rectangles were enlarged respectively.

Optical images obtained through different sensors have different resolutions and position accuracies. To validate the accuracy of the terrain slopes obtained from images taken by different sensors as auxiliary data for filtering, optical stereo images from the satellites Gaofen-7, Ziyuan-3, and Tianhui-1 were adopted to invert the terrain slopes and the correlation between the resulting slopes, and the inversion results obtained using the laser altimetry data (ATL08, dataset 1) were calculated. Previous analysis suggested that positioning errors can be associated with both optical stereo images and laser altimetry data. Moreover, the actual terrain in the study area is complex, with a wide range of land cover types. Before comparing the terrain slopes obtained using the two datasets, registration of the dense matching point clouds to laser altimetry data according to the terrain similarity theory was required to verify the correspondence between the derived slopes. The laser altimetry data describing the 4.5–22.5 km segment in dataset 1 were selected for analysis (Figure 9). The derived terrain elevations and slopes following the correlation are illustrated in Figure 10. The reference slopes were acquired by fitting the surface laser altimetry data obtained via manual inspection, while the terrain slopes were calculated using images from Gaofen-7, Ziyuan-3, and Tianhui-1, respectively. Figure 10 suggests that the terrain slopes obtained by the inversion of different types of optical stereo images could correctly reflect slope variation and are consistent with the references. However, the inversion results differed significantly from the reference slopes in some regions because of special surface features and data accuracy differences.

**Figure 11:**
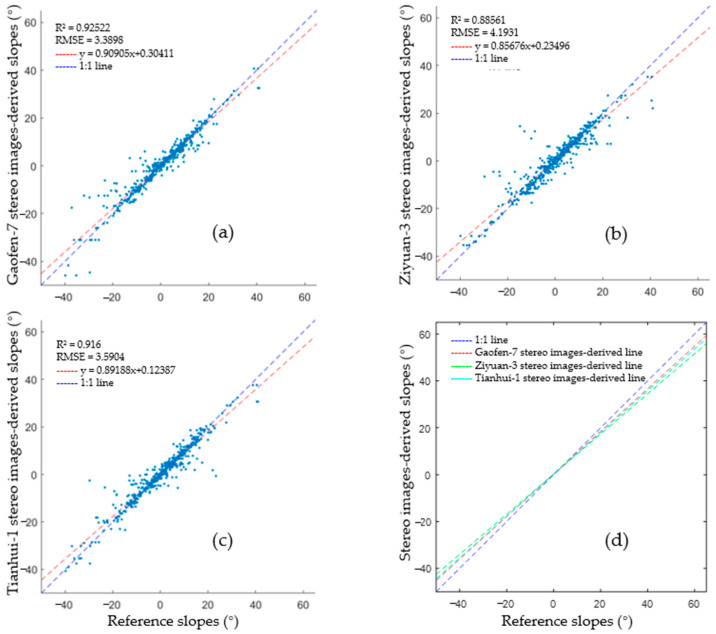
Slopes obtained from different types of optical stereo images and reference laser altimetry data. (**a**–**c**) Scatter plots of the obtained slope results, and the coefficients of determination and fitting lines of Gaofen-7, Ziyuan-3, and Tianhui-1 stereo image-derived slopes with the reference slopes, respectively. (**d**) Overlay of the three fitting lines.

According to the designed algorithm described in Section 2.2.3, when the slopes obtained from optical stereo images were used for clustering, the best-fitting slope near the signal point was adopted as the search direction in the elliptical neighborhood. Meanwhile, to consider the effects of registration and data accuracy, selecting optimal slopes from optical images for quantitative evaluation was necessary. In other words, of the slopes calculated in segments that were adjacent to the segment of interest, the slope that was closest to the reference slope was selected for comparison. Figure 11 shows the coefficients of determination and root mean square errors (RMSEs) between the slopes obtained based on various types of images and the reference data. It is clear that the slopes calculated through the inversion of different optical images vary, but all have high coefficients of determination with the reference slopes and small RMSEs in terms of the slope value. The slopes obtained from inverting the optical satellite images can thus be considered to show a strong positive correlation with the reference slopes. It is worth noting that in vegetation/building transition zones or locations in which the terrain changes (e.g., the area enclosed by green dotted rectangle in Figure 10), as well as regions with significant terrain changes (e.g., the area enclosed by blue dotted rectangle), the derived slopes may differ considerably from the reference slopes. However, it is not essential to calculate the slopes at the surface points in the laser altimetry data during the clustering process, and the best-fitting slopes in these locations can be selected. The actual RMSE should have priority over the calculated value. Hence, it is more feasible to use the slopes obtained from inverting the optical satellite images as a reference for neighborhood searching when extracting surface signal points from laser altimetry data.

### 3.2. Extraction of Typical Ground Surface Signal Photons

The laser altimetry data in the overlapping areas covered by Gaofen-7, Ziyuan-3, and Tianhui-1 (dataset 2) were adopted to evaluate the performance of the optical stereo images at different resolutions, as well as the positioning accuracy in assisting the extraction of surface signal photons. Various terrain types, including mountains and plains, with relatively significant variations are present, together with a moderate amount of vegetation and buildings, in the study area. Hence, the algorithms’ performance can be comprehensively assessed. In this study, the semi-major length of the elliptical neighborhood in the clustering algorithm was set equal to the laser spot diameter (17.5 m), and the semi-minor length was set to 3 m. With these values, the fitting accuracy of slopes from dense matching point clouds and the terrain variations could be sufficiently considered and excess noise avoided, meaning that the local densities were not underestimated. The same values were set for these two parameters in all subsequent experiments.

**Figure 12:**
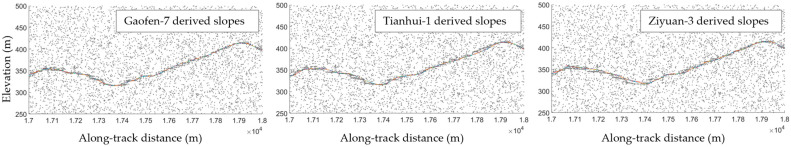
Slopes and elevations obtained using different types of optical stereo images. Gray dots represent the ATL03 data, while the colored lines denote the surface slopes and elevations obtained from the optical stereo images of Gaofen-7, Tianhui-1 and Ziyuan-3. The length of the colored lines is the same as the length of the segment, each line has a different color.

Figure 12 shows the slopes and elevations derived from the dense matching point cloud data using the three types of optical stereo images. For better illustration, the results derived at 17–18 km from dataset 2 were selected. After registration, the derived slopes and elevations from the three types of stereo images differed slightly in location with vegetation, buildings, or significant terrain variations. However, the results were generally consistent with the actual terrain and accurately reflected the changes. According to the aforementioned analysis, the derived and actual slopes may vary considerably when the elevations change abruptly in locations including features such as buildings, vegetation, and steep terrain. However, optimal selection based on segments adjacent to the segment of interest allows correct reflection of the terrain slope in a segment.

**Figure 13:**
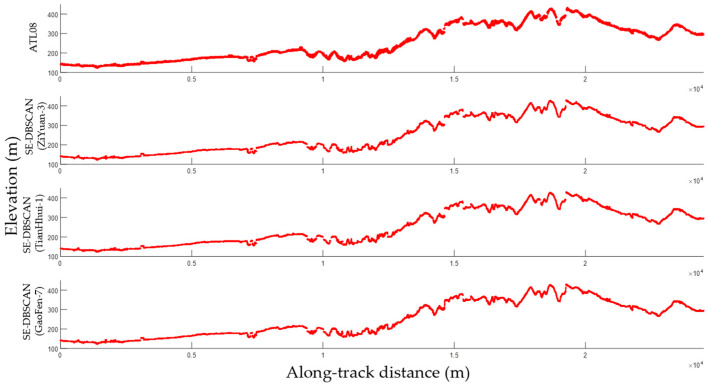
Comparison of the results from different signal photon extraction methods for a strong beam.

The signal photon extraction results of dataset 2 are shown in Figure 13. To both qualitatively and quantitatively evaluate the extraction results, the strong beam results in dataset 2 obtained using the proposed algorithm were compared with the extraction results derived using the ATL08 algorithm. According to the figure, both the proposed algorithm and the ATL08 algorithm were able to extract continuous ground surface signal photons.

The performance of the proposed and ATL08 algorithms was quantitatively analyzed using recall, precision, the *F*_1_-score (Table 3). The results demonstrated that the extracted ground surface signal photons were relatively accurate when assisted by slopes and elevations derived from the Gaofen-7, Tianhui-1, and Ziyuan-3 stereo images. The precision of the extraction results based on the three types of images was 94.58%, 94.67%, and 94.86%, respectively. The average precision of 94.70% was higher than that of the ATL08 algorithm (84.41%). However, the ATL08 algorithm achieved a higher recall (96.96%). This is mainly because, in order to extract more vegetation points, the ATL08 algorithm classifies points that are close to the ground surface as signal points; thus, more noise points are included, and precision is reduced. According to the *F*_1_-score, the proposed algorithm slightly outperformed the ATL08 algorithm when the three types of images were used.

### 3.3. Extraction of Weak Beam Signal Photons

Weak beam laser altimetry data are prone to the influence of terrain and land cover types, rendering it difficult to extract signal photons from these data. To validate the applicability of the proposed algorithm in extracting signals from weak beam laser altimetry data, dataset 3 was selected for experimentation. The data were acquired on the western side of the study area at a similar time to that of the Tianhui-1 images. Several types of terrain, including mountains and plains with substantial elevation variations, are present within the study area, along with vegetation and buildings, meaning that the algorithm performance could be fully assessed. Stereo optical images from Tianhui-1 were selected to assist in the extraction of signal photons from weak beam data. The results were compared with the extraction results obtained using the ATL08 algorithm and the true values obtained by manual inspection.

**Figure 14:**
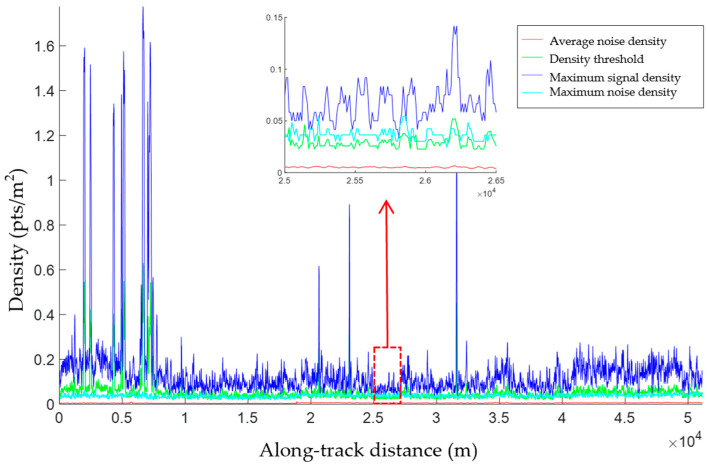
Extracted density parameters. The red, light green, blue, and green lines denote the average noise density, maximum noise density, maximum signal density, and density threshold in each segment, respectively. Picture at the top center shows the enlarged extracted result in the dashed red rectangle.

The noise density, maximum noise density, maximum signal density, and density threshold of each segment in dataset 3 are illustrated in Figure 14, together with an enlarged view of some of the results. The noise density and the maximum noise and signal densities varied significantly along the track direction. The segment-wise density threshold extracted using the proposed algorithm, which varied in the same manner, lay between the maximum signal density and the maximum noise, allowing the maximum number of noise photons to be removed. Overall, significant signal density variations were observed along the track direction, and the average and maximum noise densities were also considerable. However, the density threshold could be adjusted according to the noise level in each segment using the proposed algorithm.

**Figure 15:**
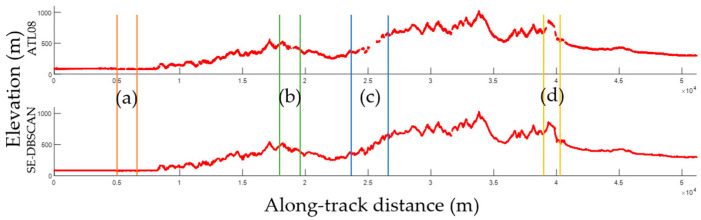
Comparison of the results from different signal photon extraction methods for a weak beam. Extraction results between orange lines, green lines, blue lines and yellow lines will be enlarged in Figure 16 for further analysis.

Figure 15 shows the final signal photon extraction results obtained using the ATL08 and proposed algorithms. The proposed algorithm was able to extract more continuous signal photons, while the ATL08 algorithm resulted in minor discontinuity at some localities (intervals bounded by vertical lines of the same color in Figure 15). Some signal photons were lost at these localities, which were situated in a steep or undulating terrain. The steep slope in these areas meant that the SNR was low, rendering signal photon extraction difficult. Continuous signals could be obtained using the proposed algorithm because the elevations and slopes derived from optical stereo images were adopted.

**Figure 16:**
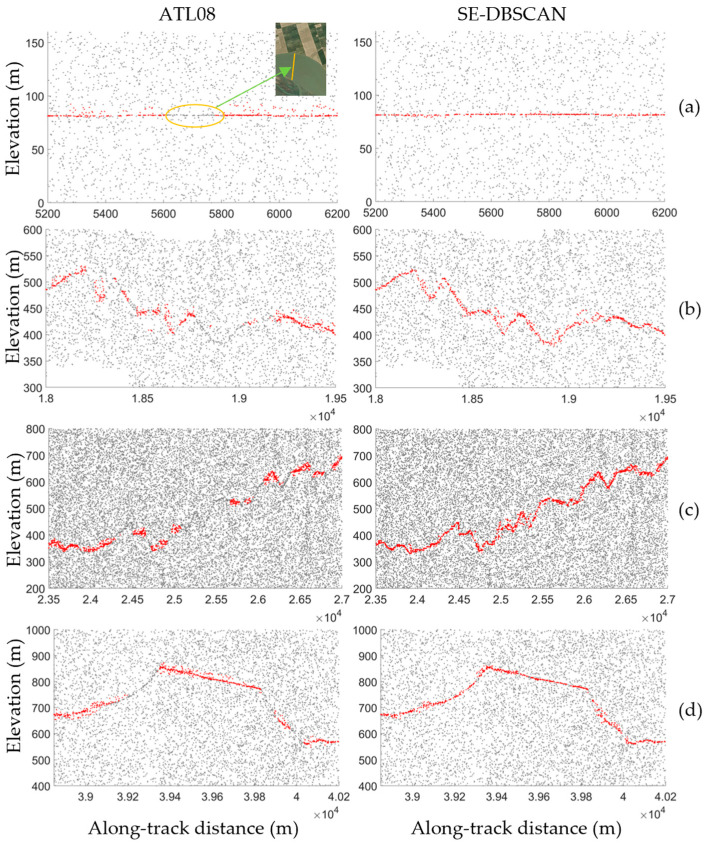
Comparison of the signal extraction results for typical areas. A flat terrain (**a**), an undulating terrain (**b**,**c**), and a steep slope (**d**) are shown. Gray dots represent the ATL03 data, while red dots represent the extraction results extracted by the ATL08 algorithm and the proposed algorithm. Yellow line in the image represents the location of surface signal points in the yellow elliptic, which were not extracted by the ATL08 algorithm.

The intervals of discontinuity in Figure 15 are enlarged in Figure 16. Figure 16a shows a flat terrain with significant noise density variations. The ATL08 algorithm resulted in signal loss at localities with abrupt density changes, while the proposed algorithm successfully extracted continuous signals. Furthermore, the ATL08 algorithm classified photons close to the ground surface as vegetation points. However, the specified area was actually a wide water body and winter farmland, with no vegetation. Meanwhile, continuous and reliable ground surface signal photons were extracted by the proposed algorithm. Figure 16b,c present the extraction results for an undulating terrain. The ATL08 algorithm was unable to extract signal points for some terrain segments, whereas continuous ground surface signals that more accurately reflect the undulating terrain were obtained using the proposed algorithm. Finally, the extraction results for a steep slope are shown in Figure 16d. The terrain varied significantly, with a maximum slope greater than 40°. The ATL08 algorithm lost ground surface signals over approximately 200 m on two sides of the slope; however, with the help of the elevations and slopes derived from optical stereo images, better extraction results were obtained for the steep slope using the proposed algorithm and a continuous terrain was acquired.

**Figure 17:**
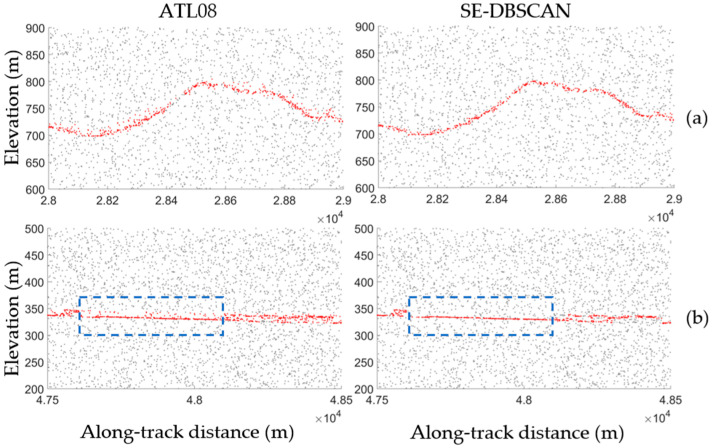
Comparison of the extraction results for typical ground features. Land cover with low vegetation (**a**) and buildings (**b**) are shown. Gray dots represent the ATL03 data, while red dots represent the extraction results extracted by the ATL08 algorithm and the proposed algorithm. The dashed blue rectangle shows an area adjacent to a building.

In regions with complex land cover types, such as low vegetation (Figure 17a) or buildings (Figure 17b), complete signals of ground features were obtained by both the proposed algorithm and ATL08. However, the latter wrongly classified noise photons that lay close to the ground surface as surface signals. In addition, for a 500 m-long area to the left of the building (area bound by a dashed rectangle in Figure 17b), the ATL08 algorithm used the building height as a reference (10 m) and included all photons close to the ground surface in the signal extraction results, leading to the inclusion of more noise points.

The performance of the proposed algorithm and ATL08 was quantitatively analyzed using recall, precision, and the *F*_1_-score (Table 4). A recall of 89.42% was obtained by ATL08, which was higher than that derived by the proposed algorithm (86.03%). However, the precision of the former (87.82%) was lower than that of the latter (92.68%). To extract more vegetation points, the ATL08 algorithm classified points close to the ground surface as signal points. Hence, the recall was higher; however, noise points that were close to the ground surface were wrongly identified as signal points, reducing the precision. According to the aforementioned analysis, signal loss was noted for steep slopes when using ATL08, while relatively continuous terrains could be derived using the proposed algorithm. The data used in evaluating the proposed algorithm covered 51 km and the two long steep slopes with signal loss were only 139 m and 70 m long, meaning that the associated signal loss accounted for only a small proportion of the total length. Therefore, the final precision of the proposed algorithm could be considered slightly better than that of ATL08, and the *F*_1_-scores of the two algorithms were similar (approximately 89%).

The ATL08 algorithm did not generate satisfactory results for steep slopes because the pulse width was stretched on steep slopes and there were fewer photons per unit area compared to flat terrains. However, the proposed algorithm used local density thresholds and best-fitting slope estimation based on dense matching point clouds to effectively determine the optimal direction for the neighborhood search, allowing better signal photon extraction results to be attained. The weak beams of the laser altimeter systems had fewer measurement points in areas with sparse vegetation and low shrubs, meaning that the overall density of the measurement points was low. To obtain reliable ground surface points, the proposed algorithm did not identify photons close to the ground surface as signal points, striking a balance between recall and precision; thus, the proposed algorithm was able to successfully obtain continuous terrains from the weak beam ICESat-2 data in mountainous areas even when the SNR was typically very low.

### 3.4. Limitations and Recommendations

Due to the revisited period and cloud cover, it was difficult to obtain high-quality optical stereo images that are completely consistent with the acquisition time of laser altimetry data in the study area. Cloud cover in images may result in holes during dense matching, and some measures need to be taken to reduce the impact of the cloud, such as atmospheric correction or filling the holes with dense matching point clouds generated from other optical stereo images. Moreover, if the acquisition time of optical stereo images and laser altimetry data is different, the ground surface may undergo some changes, and the derived slopes may be different from those of the current ground surface. Although our proposed optimal slope selection method can help reduce the effect of slope estimation error, some uncertainty may still exist. Thus, the acquisition time of optical stereo images and laser altimetry data should be as close as possible.

## 4. Conclusions

This study proposed an adaptive signal photon extraction method that combines slope and elevation information derived from optical stereo images. Signal photon extraction was performed for laser altimetry data in cases with different land cover types and terrains. The experimental results demonstrated that the proposed algorithm could adjust the search direction and the density threshold for classification according to the along-track slope and the signal photon density. The proposed algorithm was found to be highly adaptive to laser altimetry data of various terrains taken at different times or beam energy levels. It could effectively and accurately extract ground surface signal photons using data from both strong and weak beam photon-counting laser altimeter systems, allowing the generation of continuous ground surface profiles. Even in cases where the SNR was low, i.e., when weak beam data acquired in the daytime were used, the proposed algorithm could extract relatively reliable and continuous signal photons from the ground surface. 

This is the first study in which slope and elevation information obtained from optical stereo images was adopted to assist in extracting ground surface signal photons from ICESat-2/ATLAS data. This research proves the valuable support and important role of information from optical stereo images in ICESat-2/ATLAS signal extraction. Effective solutions are available for extracting ground surface signal photons from ICESat-2/ATLAS data. In this study, a plane fitting-based strategy to extract the along-track slope, a registration strategy based on initial laser altimetry data, and an optimal slope selection strategy were designed, which successfully solve issues associated with the low positioning accuracy of optical stereo images, undulating terrains, and complex and diverse land cover types where optical images are adopted to assist in signal photon extraction from laser altimetry data. Hence, accurate directions were attained for signal search and clustering. This was further combined with local thresholding to significantly reduce the dependence of the algorithm on the included parameters and increase its robustness. Furthermore, the present study also achieved composite processing of optical images and laser altimetry data to lay a good research foundation for improving the positioning of optical images. 

Future research may focus on extracting reliable information using optical stereo images from different sources to assist in the effective extraction of ground surface signal photons from ICESat-2/ATLAS data for complicated surface environments and, in turn, improve the positioning of optical stereo images.

## Figures and Tables

**Table 1:** The main parameters of the images in the study area.

Satellite	Launch Country	Launch Time	Spatial Resolution	Geopositioning Accuracy without GCPs
Gaofen-7	China	2019	Front view: 0.8 m Back view: 0.65 m	Horizontal: 6 m Vertical: 2 m
Ziyuan-3	China	2016	Front view: 2.5 m Ndir: 2.1 m Back view: 2.5 m	Horizontal: 10 m Vertical: 8 m
Tianhui-1	China	2015	Front view: 5 m Ndir: 4.1 m Back view: 5 m	Horizontal: 7 m Vertical: 5 m

**Table 2:** Data overview of the study area.

Data Source	Name	Acquisition Time
Gaofen-7	GF701_006787_E113.4_N34.6_20210122113017_BWD_01_ SC0_0001_2101256512.tif GF701_006787_E113.4_N34.6_20210122113017_FWD_01_ SC0_0001_2101256512.tif	22 January 2021
Ziyuan-3	zy301a_bwd_045459_006138_20200317111756_01_ sec_0001_2003183114.tif zy301a_nad_045459_006138_20200317111727_01_ sec_0001_2003186186.tif	17 March 2020
Tianhui-1	TH01-03_P202101189046321_1B_SXZ_1_08_005_135.tif TH01-03_P202101189046321_1B_SXZ_3_08_005_135.tif	18 January 2021
ATL03/ATL08	ATL03_20200829230935_10020802_005.h5 (gt3l, dataset 1) ATL08_20200829230935_10020802_005.h5 (gt3l, dataset 1)	29 August 2020
	ATL03_20210218030147_08571006_005.h5 (gt3r, dataset 2) ATL08_20210218030147_08571006_005.h5 (gt3r, dataset 2)	18 February 2021
	ATL03_20201222054939_13600906_005.h5 (gt1l, dataset 3) ATL08_20201222054939_13600906_005.h5 (gt1l, dataset 3)	22 December 2020

**Table 3:** Quantitative evaluation of the algorithms’ performance using the strong beam dataset. Gaofen-7, Tianhui-1, and Ziyuan-3 used the SE-DBSCAN algorithm.

Algorithm	Total Number	*TP*	*FN*	*FP*	*R*	*P*	*F*_1_
ATL08	30,119	25,424	797	4695	96.96%	84.41%	90.25%
Gaofen-7	24,962	23,608	2613	1354	90.03%	94.58%	92.25%
Tianhui-1	24,606	23,295	2926	1311	88.84%	94.67%	91.66%
Ziyuan-3	24,286	23,038	3183	1248	87.86%	94.86%	91.23%

**Table 4:** Quantitative evaluation of the algorithms’ performance using the weak beam dataset.

**Algorithm**	**Total Number**	***TP***	***FN***	***FP***	***R***	***P***	***F*_1_**
ATL08	31,443	27,613	3267	3830	89.42%	87.82%	88.61%
SE-DBSCAN	28,664	26,567	4313	2097	86.03%	92.68%	89.23%

## Data Availability

Publicly available datasets were analyzed in this study. ICESat-2 data were downloaded from the NASA National Snow and Ice Data Center (NSIDC) (https://nsidc.org/data/icesat-2 (accessed on 5 January 2023)).
